# The Effect of Preoperative Type 2 Diabetes and Physical Fitness on Mental Health and Health-Related Quality of Life after Roux-en-Y Gastric Bypass

**DOI:** 10.1155/2016/3474816

**Published:** 2016-06-09

**Authors:** Cathrine L. Wimmelmann, Michael T. Lund, Merethe Hansen, Flemming Dela, Erik L. Mortensen

**Affiliations:** ^1^Section of Environmental Health, Department of Public Health, University of Copenhagen, 1353 Copenhagen K, Denmark; ^2^Center for Healthy Aging, Faculty of Health and Medical Sciences, University of Copenhagen, 2200 Copenhagen N, Denmark; ^3^Xlab, Department of Biomedical Sciences, University of Copenhagen, 2200 Copenhagen N, Denmark

## Abstract

*Objective*. To investigate the predictive value of type 2 diabetes and lack of physical activity for mental health and health-related quality of life after Roux-en-Y gastric bypass.* Method*. Forty severely obese patients undergoing Roux-en-Y gastric bypass were included in the GASMITO study. Information about physiological and psychological factors was prospectively assessed at four time points, two times prior to surgery and two times after surgery. Measures included oral and intravenous glucose tolerance tests, VO_2_max test,* Symptoms Checklist (SCL-90), Short Form Health Survey 36 (SF-36), Body Image Questionnaire*, and a questionnaire assessing sociodemographic factors and medical status.* Results*. Mean % excess weight loss was 65% (±12) at 18-month follow-up and 50% of the participants with diabetes experienced total remission. Also, significant improvements were observed with regard to physical fitness, mental distress, health-related quality of life, and weight-related body image (*p* < 0.05). The interaction between follow-up time and type 2 diabetes at baseline significantly predicted six of the thirteen psychological subscales (*p* < 0.05) and, across the follow-ups, physical fitness level made modest contributions to variations in mental symptoms and HRQOL but not weight-related body image.* Conclusion*. The results suggest that baseline difference in mental symptoms and physical HRQOL between diabetic and nondiabetic patients declines across follow-ups and resolves around the time of surgery.

## 1. Introduction

Bariatric surgery is the most effective treatment for severe obesity often resulting in excess weight loss of 50–80% [1, 2] and improved psychological wellbeing and health-related quality of life (HRQOL) [3–5]. In addition, particularly, Roux en-Y gastric bypass (RYGB) has been shown to lead to remission of type 2 diabetes [6] and consequently many severely obese individuals with type 2 diabetes seek out this surgical procedure. However, results vary and 7%–50% of patients undergoing bariatric surgery do not achieve a successful weight loss (% EWL < 50) or regain weight when the surgical effects begin to abate [7–9]. A growing body of research has suggested that emotional and psychological impairments after surgery influence or may even be the cause of the suboptimal weight loss and weight regain in some bariatric patients [10, 11]. Before surgery, bariatric patients report elevated levels of mental distress and reduced HRQOL [12] compared with the general and nonsurgical obese population. Also, in bariatric populations, the prevalence of current psychiatric disorders has been found to be as high as 56% and up to 73% report to have had at least one psychiatric disorder at some point in their life [13]. In defining a successful treatment outcome after bariatric surgery, mental health and HRQOL are therefore equally important success criteria as weight loss. Unfortunately, the importance of postoperative psychological factors has often been overlooked in the bariatric literature and further research is highly needed.

Type 2 diabetes and lack of physical activity in bariatric patients have previously been associated with decreased weight loss after surgery [14–16]. However, the predictive value of preoperative medical comorbidities and behavioural factors including type 2 diabetes and physical activity, for mental health and HRQOL, is currently unclear [17]. It is well established that medical comorbidities such as type 2 diabetes are associated with impaired mental health and HRQOL both in the general and in the nonsurgical obese population [18–20]. For instance, a large meta-analysis of 42 studies with a combined total of 20.218 subjects concluded that individuals with diabetes have twice the risk of depression compared with nondiabetic individuals [18]. However, in a recent study, we investigated mental health and HRQOL in 129 RYGB candidates and found indications that the relation between type 2 diabetes and mental health may differ in the bariatric population [21]. More specifically, results from this study showed that bariatric patients with type 2 diabetes presented for RYGB surgery with better mental health and HRQOL compared with nondiabetic patients, especially with regard to physical aspects of HRQOL. These results suggest that, in our study, patients with type 2 diabetes sought out the surgical option at an earlier stage than the nondiabetic patients, primarily to target their diabetes. In contrast, the nondiabetic patients may have experienced severe mental and physical impairments before considering surgical obesity treatment. Despite the observed psychological differences between patients with and without type 2 diabetes, no studies have investigated the predictive value of baseline type 2 diabetes for the mental health and HRQOL after bariatric surgery.

In addition, lack of physical activity has been associated with poor mental health and reduced HRQOL in the bariatric population [22]. In a large cohort of severely obese adults, King et al. [22] found an inverse association between physical activity and mental health prior to undergoing bariatric surgery. Furthermore, a study with 131 bariatric patients reported that both frequency and intensity of physical activity one year after RYGB were independently associated with better mental HRQOL and fewer depressive symptoms [23]. These results suggest that the lack of physical activity may negatively predict mental health and HRQOL after bariatric surgery. However, to our knowledge, no studies have prospectively investigated the effect of physical activity on mental health among bariatric patients. Furthermore, while these studies provide some evidence of an association between physical activity and psychological wellbeing among bariatric patients, the majority of the research relies on self-reported physical activity [23–25] and no studies have investigated the relation using an objective measure of the patients' actual physical fitness level.

To address these shortcomings in the literature, the aim of this study was to prospectively investigate the effect of objectively measured baseline type 2 diabetes and physical fitness level on mental health, HRQOL, and weight-related body image after RYGB.

## 2. Methods

### 2.1. Design

The current study is part of the larger GASMITO study (the National Committee on Health Research Ethics, study protocol number HC-2009-050) that prospectively investigates psychological and physiological factors in severely obese RYGB patients with and without type 2 diabetes from the time before weight loss is started until weight stability is achieved 1.5 years after RYGB. All patients were tested at four follow-ups: (A) at baseline, prior to weight loss, (B) about two months later after a diet induced weight loss, (C) 4 months after RYGB, and, finally, (D) 18 months after RYGB. Each test session consisted of three test days which included a number of physiological tests including oral and intravenous glucose tolerance tests, 2-hour hyperinsulinemic euglycemic clamp, dual X-ray absorptiometry scan, and incremental bicycle VO_2_max test. Furthermore, the psychological profile of the patients was assessed using a battery of standardized questionnaires including measures of mental symptoms, HRQOL, body image, and lifestyle, which were administered at each of the four follow-ups.

### 2.2. Sample

In total, 40 GASMITO participants completed the psychological and physiological tests at baseline, 32 participants at follow-up (B), 31 participants at follow-up (C), and finally 23 participants at follow-up (D). The main reason for dropout was the time associated with the multiple tests days and some tests were furthermore associated with some pain or discomfort. Participants were sampled consecutively at the bariatric clinic at Hamlet Hospital or at Køge Hospital, two hospitals located in Copenhagen and suburban Copenhagen area, respectively. Participants were contacted at their first presurgical consultation at the hospital or contacted over the phone after their initial consultation and invited to an information meeting before inclusion. All GASMITO participants were informed verbally and in writing about the procedures and were asked to sign a consent form before being included in the study. Inclusion criteria were in agreement with the Danish guidelines for gastric bypass surgery prior to January 2011, which allowed individuals between 18 and 60 years of age with a BMI > 40 kg/m^2^ or BMI > 35 kg/m^2^ and obesity-related comorbidities (e.g., type 2 diabetes) to be considered for surgery. Exclusion criteria for the current study comprised heart disease, renal disease, or hyper- or hypothyreosis.

### 2.3. Measures and Tests

To assess mental symptoms, a short version of the Danish* “Symptoms Checklist” (SCL-90)* [26] was used. This 90-item checklist assesses the severity of a wide range of mental symptoms and is scored on a scale ranging from 0 to 4 with 4 reflecting the highest level of symptom load. Only the three subscales (35 items) measuring somatization, depression, and anxiety were used, as these symptoms are expected to be prevalent among the patients. Furthermore, the General Severity Index (GSI), which reflects both number and severity of symptoms, was calculated as the mean score of all 35 items.

To assess quality of life and general health status of the patients, we used the* “Short Form Health Survey 36” (SF-36)* [27], which consists of 36 items evaluating eight aspects of HRQOL including physical function, physical role, physical pain, general health, vitality, social function, mental role, and mental wellbeing. Raw scores from the SF-36 were transformed to scores ranging from 0 to 100 with 100 indicating the highest level of HRQOL.

Information about the patients' background, lifestyle, and medical status was obtained using a short version of the questionnaire that was developed as part of the* Copenhagen Aging and Midlife Biobank (CAMB)* [28]. This questionnaire comprises 76 questions providing information on social background, social situation, lifestyle, and physical health status.

The* Body Image Questionnaire (BIQ)* [29] was used to assess the patients' body image and comprises 50 items scored on a scale from 1 to 5, with 5 indicating a more positive body image. A mean item score for “weight-related items only” was calculated as the sum of items 3, 15, 19, 24, 27, 30, 31, 34, 40, 45, 48, and 50 divided by 12. This score was expected to be especially sensitive to weight-related changes in body image.

Diabetes status was determined by oral and intravenous glucose tolerance tests. The plasma samples were cooled and centrifuged at 2000 ×g for 10 minutes before the plasma was stored at −80°C until analysis was conducted. Remission of type 2 diabetes was defined as HbA1c < 42.1 mmol mol^−1^, fasting glucose < 5.6 mmol L^−1^, and no medication.

The VO_2_max (mL/min/kg) is an objective measure of physical fitness level and a direct indicator of the extent of physical activity. Participants performed an incremental bicycle VO_2_max test on a stationary bike until exhaustion. A test was considered valid when leveling off was achieved, which was defined as unchanged oxygen uptake despite increasing work load, and when a respiratory exchange ratio greater than 1.15 was observed. Maximal oxygen uptake was calculated as the highest average uptake during 20 seconds of exercise.

Finally, a dual X-ray absorptiometry scan (DXA scan) was performed to examine the body composition and body fat distribution of the patients.

### 2.4. Data Analysis

Descriptive statistics were used to investigate patient characteristics at baseline. To avoid losing information due to missing follow-up data points, we analysed the repeated data using a random intercept multilevel model, allowing intercepts to vary between subjects, with within-subjects level 1 (follow-up time) and between-subjects level 2 with the subjects GASMITO ID as grouping variable for the measures. The first-order autoregressive covariance structure was used. All analyses of the repeated data were performed using the GENLINMIXED procedure in SPSS 20.0 with the robust standard error option to accommodate nonnormality in some of the outcome variables. To investigate the effect of baseline diabetes and physical fitness level on the psychological outcome variables, the multilevel model included follow-up time, sex, age, baseline BMI, baseline VO_2_max, baseline diabetes, and their interaction with follow-up time as fixed effects covariates. Age, BMI, and VO_2_max at baseline were included as continuous variables in the interaction analyses with follow-up time.

## 3. Results

### 3.1. Patient Characteristics at Baseline

In total, 40 bariatric patients were included in the GASMITO study, 30% (*n* = 12) men and 70% (*n* = 28) women. The participants' mean age at baseline was 39.5 (±8.9) years, their mean BMI was 42.5 (±4.5) kg/m^2^, and their mean VO_2_max was 21.2 (±4.7) mL/kg/min, reflecting a very poor to poor level of physical fitness [30]. Finally, 35% (*n* = 14) of the participants had type 2 diabetes at baseline and 65% (*n* = 26) were classified as nondiabetic. Patients with and without diabetes had similar age, weight, BMI, and VO_2_max prior to surgery. However, body fat % was lower in the diabetic than in the nondiabetic patients (45.1% (±5.9) versus 49.6% (±5.5), *p* = 0.024).

### 3.2. Weight Loss and Change in Mental Health across Follow-Ups

Table 1 shows the baseline results for weight, BMI, VO_2_max, mental symptoms (SCL-90), HRQOL (SF-36), and weight-related body image (BIQ) and the difference in each subscale score from one follow-up to another.

Weight and BMI improved significantly in the bariatric patients. These weight-related improvements were highly significant from baseline to follow-up (B) immediately before surgery and continued to be highly significant throughout the surgical course. At follow-up (D), 18 months after RYGB, the mean percent excess weight loss (% EWL) from baseline reached 65% (±12) and percent weight loss (% WL) was 30% (±6), indicating a successful surgical weight loss outcome [31]. Patients with and without type 2 diabetes at baseline did not differ significantly with regard to % EWL. At 18-month follow-up, the two groups reached an EWL of 63.0% and 66.0%, respectively. Furthermore, of the 14 patients with baseline type 2 diabetes, 50% (*n* = 7) experienced total remission after surgery and only 21% (*n* = 3) clearly met the criteria for diabetes (HbA1c > 48) postoperatively. Also, VO_2_max increased significantly after surgery possibly explained by the decreased body mass of the patients.

At 18-month follow-up, the bariatric patients reported a significant reduction of mental symptoms and a significant increase in HRQOL and weight-related body image compared with baseline values, indicated by lower scores on the somatization, depression, anxiety, and GSI subscale of the SCL-90 and higher scores on the eight SF-36 subscales and the weight-related BIQ subscale (*p* < 0.05 for all subscales). As Table 1 shows, the improvements were evident in most of the SCL-90 and SF-36 subscales from the time immediately before surgery (follow-up (B)) to four months after surgery (follow-up (C)) with the most pronounced improvements observed on the physical HRQOL subscales. Of the SCL-90 and SF-36 subscales, only somatization and social function did not improve during the first four postoperative months. Furthermore, the somatization, depression, GSI, physical function, vitality, and social function subscales improved postoperatively until 18 months after surgery. Finally, the weight-related body image score increased significantly throughout the surgical course and was, together with physical function, the only subscale demonstrating significant changes at each follow-up.

### 3.3. Effects of Type 2 Diabetes on Mental Health, HRQOL, and Body Image

**Figure 1:**
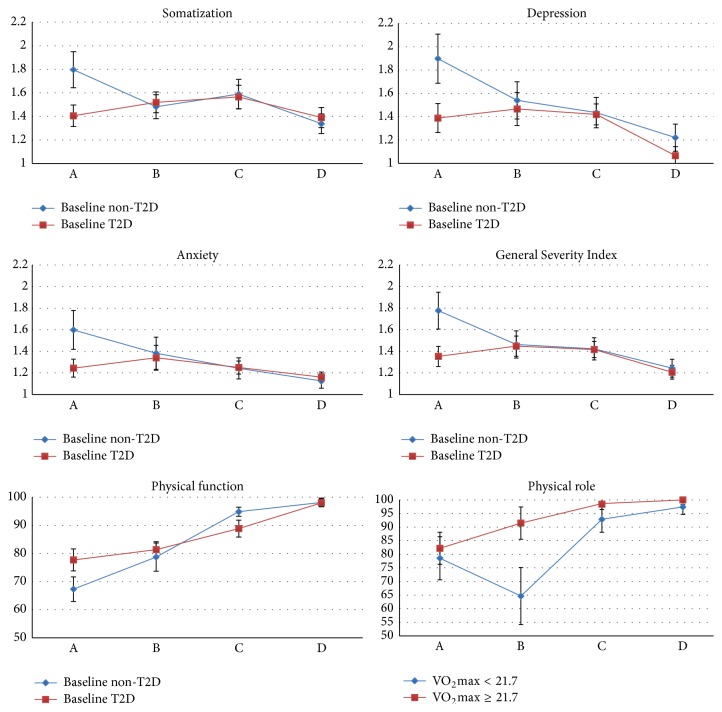
Estimated means and standard errors (bar) across follow-ups for patients with and without type 2 diabetes at baseline. Only subscales with a significant interaction effect of type 2 diabetes and follow-up are illustrated. The somatization, depression, anxiety, and General Severity Index (GSI) subscales of the “Symptoms Checklist” (SCL-90) are scored from 1 to 4 with higher scores indicating more mental distress. The physical function and physical role subscales of the “Short Form Health Survey 36” (SF-36) are scored from 1 to 100 with higher scores reflecting better health-related quality of life.

In the applied multilevel model, the interaction between follow-up time and type 2 diabetes at baseline was found to be significant for six of the thirteen investigated subscales after adjusting for main effects of sex, age, baseline BMI, baseline VO_2_max, and their interaction with follow-up time.  Figure 1 shows the effect of baseline diabetes across follow-ups for the six subscales where this interaction was found to be significant.

The only significant main effect of baseline diabetes was observed for the emotional role subscale of the SF-36 (*p* = 0.011). However, the interaction between follow-up time and diabetes was significant for the somatization (*p* = 0.005), the depression (*p* = 0.004), the anxiety (*p* = 0.036), and the GSI (*p* = 0.001) subscales of the SCL-90. In addition, the interaction between follow-up time and diabetes was significant for the physical function (*p* = 0.002) and the physical role (*p* = 0.016) subscales of the SF-36. As illustrated by Figure 1, these results indicate that differences in mental distress and physical aspects of HRQOL between patients with and without baseline diabetes were only substantial at baseline assessment, but not at the later follow-ups. No significant main or interaction effects of diabetes were observed for the weight-related body image subscale.

### 3.4. Effects of Physical Fitness on Mental Health, HRQOL, and Body Image

**Figure 2:**
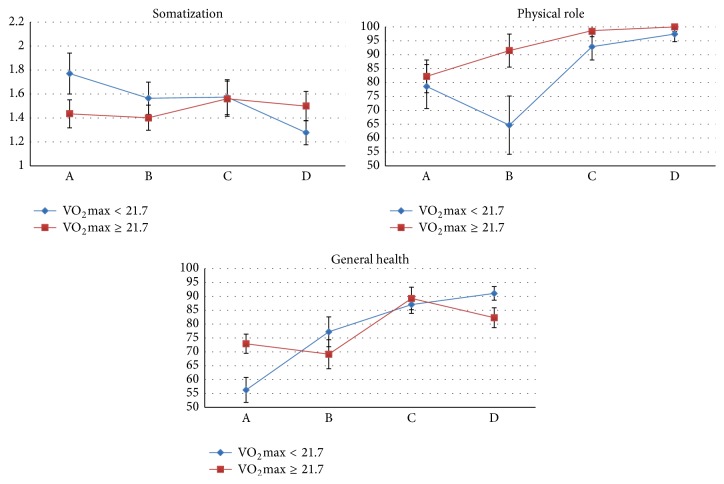
Estimated means and standard errors (bars) across follow-ups for patients with a baseline physical fitness level lower and higher than the sample median (VO_2_max = 21.7 mL/min/kg). Only significant interaction effects of physical fitness level and follow-up are illustrated. The somatization subscale of the “Symptoms Checklist” (SCL-90) is scored from 1 to 4 with higher scores indicating more mental distress. The physical role and general health subscales of the “Short Form Health Survey 36” (SF-36) are scored from 1 to 100 with higher scores reflecting better health-related quality of life.

Physical fitness (VO_2_max) made modest contributions to variations in mental symptoms and HRQOL but not weight-related body image. Main effects of VO_2_max were observed for the physical function (*p* = 0.031), the physical role (*p* = 0.015), and the vitality (*p* = 0.018) subscales of the SF-36 with higher baseline VO_2_max associated with higher scores on these subscales. Also, the interaction between follow-up time and baseline VO_2_max was significant for the somatization (*p* = 0.000) subscale of the SCL-90 and the physical role (*p* = 0.016) and general health (*p* = 0.024) subscales of the SF-36 suggesting that the effect of baseline physical fitness level on these subscales declines after surgery. To illustrate this, baseline VO_2_max was recoded into a binary low-high variable using the median (VO_2_max = 21.7 mL/min/kg) to distinguish the two groups.  Figure 2 shows the effect of baseline physical fitness level on the three subscales, somatization, physical role, and general health, across the four follow-ups.

### 3.5. Other Significant Predictors

Several significant predictors were observed in addition to baseline diabetes and baseline physical fitness. More specifically, the main effect of sex was significant for physical function (*p* = 0.030) and weight-related body image (*p* = 0.004) with women demonstrating lower scores than men on these subscales. The interaction between follow-up time and sex was significant for the depression (*p* = 0.005) score of the SCL-90 and the physical function (*p* = 0.015), the general health (*p* = 0.024), the vitality (*p* = 0.032), and the mental health (*p* = 0.010) scores of the SF-36. In general, these interactions reflected that sex differences tended to become smaller across follow-ups. However, for the vitality and the mental health subscale of the SF-36, the direction of the association between sex and these subscale scores changed after surgery and sex differences remained at 18-month follow-up with women reporting higher scores than men. Furthermore, a main effect of age was observed for the somatization (*p* = 0.028), the depression (*p* = 0.007), and the GSI (*p* = 0.010) subscales with younger patients showing more mental distress than their older counterparts. The interaction between follow-up time and age was significant for the somatization (*p* = 0.007), the depression (*p* = 0.002), the GSI (*p* = 0.015), the vitality (*p* = 0.001), the mental health (*p* = 0.016), and the weight-related body image (*p* = 0.048) scores indicating that in general the effect of age on these subscales declines across follow-ups. Baseline BMI was of limited predictive value and only showed significant main effects for weight-related body image (*p* = 0.040) where a lower BMI at baseline was associated with higher body image scores. Finally, the interaction between follow-up time and baseline BMI was significant for the vitality (*p* = 0.000) and the mental health (*p* = 0.000) subscales suggesting that the effect of baseline BMI on these two subscales decreases from follow-up (A) to follow-up (D).

## 4. Discussion

To our knowledge, this study was the first study to prospectively investigate the importance of type 2 diabetes and physical fitness at baseline for mental health, HRQOL, and weight-related body image among bariatric patients undergoing RYGB. In line with consistent findings from prior research [4, 10], mental health, HRQOL, and weight-related body image improved significantly from preoperative levels. Improvements were most pronounced four months after RYGB particularly in physical aspects of HRQOL and weight-related body image.

### 4.1. The Importance of Preoperative Type 2 Diabetes for Mental Health and HRQOL Outcome

The interaction between follow-up time and baseline diabetes was significant for mental health and physical HRQOL outcomes indicating that the difference in mental symptoms and physical HRQOL between diabetic and nondiabetic patients declines across follow-ups. Diabetic patients endorsed fewer mental symptoms and had a higher HRQOL and better weight-related body image preoperatively compared with nondiabetic patients. This indicates that diabetic patients seeking RYGB may be motivated by their diabetes in contrast to nondiabetic patients that may seek out the surgical option due to substantial obesity-related distress. However, interestingly, the differences in mental symptoms and HRQOL between patients with and without diabetes at baseline resolved around the time of surgery. This also suggests that the psychological impairments in the nondiabetic patients may be related to the obese state and therefore immediately influenced by weight loss. It could be speculated that positive expectations to the operation outcome generate an experience of optimism that positively affects the level of mental symptoms and HRQOL in the nondiabetic patients around the time of surgery. In fact, improvements in mental health and HRQOL were more pronounced initially after surgery in patients that did not have preoperative diabetes compared with patients with diabetes at baseline in spite of the fact that diabetes resolved in 50% of these patients after surgery. One possible explanation is that the differences in psychological wellbeing between patients with and without diabetes reflect preoperative differences in weight-related mental health and HRQOL. Prior research has suggested that type 2 diabetes is not associated with major impairments of mental and physical functioning among bariatric candidates [21]. In contrast, nondiabetic patients report marked impairments in psychological and physical wellbeing prior to surgery indicating that nondiabetic patients seek out bariatric surgery at a more debilitating stage of the obese state [21]. Thus, as suggested, it is possible that bariatric candidates with diabetes primarily seek out surgery to target their diabetes, whereas some bariatric patients without diabetes at baseline seek surgery because of impaired mental health and HRQOL related to obesity. It could therefore be speculated that nondiabetic patients experience a major relief when undergoing weight loss and surgery and this might intensify the improvements in mental health and HRQOL among these patients initially after surgery. This explanation is supported by a study that investigated motivational factors among 208 gastric banding patients [32]. Interestingly, this study found that patients who sought bariatric surgery due to medical comorbidities, primarily hypertension and type 2 diabetes, had fewer depressive symptoms and higher HRQOL scores compared with patients who sought surgery to improve other factors including appearance, physical fitness, and physical limitations. However, research investigating the influence of preoperative diabetes on mental health and HRQOL after bariatric surgery is currently lacking and should therefore be investigated further.

### 4.2. The Importance of Preoperative Physical Fitness Level for Mental Health and HRQOL Outcome

Significant increases in physical activity have consistently been reported in prior research [23, 25]. These studies have mainly used self-reported physical activity with the risk for patients to overreport their physical activity due to misperceptions or reporting bias. Among the bariatric patients in our study, VO_2_max, an objective measure of physical fitness, increased significantly after RYGB. However, preoperative level of physical fitness did not contribute to the differences between diabetic and nondiabetic patients and VO_2_max was only a main predictor of improvements in the physical function, the physical role, and the vitality subscale of the SF-36 with higher VO_2_max associated with better physical HRQOL. Furthermore, the interaction between follow-up time and VO_2_max was only significant for the somatization subscale of the SCL-90 and the physical role and general health subscales of the SF-36. This may seem surprising since prior research has found that physical activity strongly predicts weight loss outcome [14, 25], fewer depressive symptoms, and higher mental HRQOL one year after RYGB [23]. However, in contrast to the majority of this research, we used an objective measure as indicator of physical activity. VO_2_max depends on body mass that is why the observed increase in physical fitness level of our patients may reflect weight loss rather than increased physical activity. Thus, with regard to the predictive value of physical activity, the use of different assessment methods may therefore explain the inconsistency between our results and prior findings.

### 4.3. The Importance of Demographic Factors for Mental Health and HRQOL Outcome

Finally, numerous attempts have been made to identify demographic predictors of weight loss after bariatric surgery with conflicting results [33, 34]. Furthermore, the effect of demographic factors including sex and age on psychological outcome after bariatric surgery is understudied and currently unclear. In line with preoperative studies [35–37], our results showed that women presented for surgery with more mental distress, reduced HRQOL, and poorer weight-related body image compared with men. Also, younger patients tended to report worse psychological wellbeing compared with their older counterparts. A more positive weight-related body image was observed among patients with lower baseline BMI compared with the patients with a higher BMI. This effect of sex, age, and baseline BMI on mental distress, HRQOL, and weight-related body image declined throughout the surgical course for most of the subscales. However, sex differences were observed on two aspects of HRQOL 18 months after surgery with women reporting higher scores on the vitality and the mental health subscales of the SF-36. Thus, in terms of HRQOL, it may seem that women benefit the most from surgery. In contrast, also using the SF-36, Masheb et al. [38] investigated postoperative HRQOL among 137 patients undergoing gastric bypass surgery and found that male sex predicted better mental HRQOL 12 months after surgery. However, this study did not adjust for diabetes status or physical fitness level. Also, due to the relatively modest number of men participating in the current study, it is difficult to draw sound conclusions about the effect of sex on postoperative HRQOL and further research is therefore highly needed.

### 4.4. Limitations

The current study is an important contribution to the very limited literature investigating type 2 diabetes and psychological outcome after bariatric surgery. The prospective study design and the use of objective methods to assess weight, BMI, type 2 diabetes, and physical fitness are important strengths of the study. In addition, the assessment of four psychological factors contributes to a fairly detailed psychological profile of the bariatric patients. However, some limitations should be mentioned. The relatively small sample size, questioning the power of the study, and the lack of comparison with a nonsurgical obese control group are both limitations of the study. Thus, significant findings may have been missed and, furthermore, it remains unclear whether the results are specific to the bariatric population or whether they also apply to the severely obese individuals in traditional weight loss programs. Also, though prospective, by design, the study was correlational. Thus, it is not possible to infer causality and therefore not possible to examine whether baseline type 2 diabetes or baseline fitness level has causal effects on psychological outcome after RYGB surgery. Finally, patients were followed up to 18 months to two years after their operation. However, the American Society for Bariatric Surgeons has recommended research to conduct at least 5-year follow-ups and the results of the current study should therefore be considered preliminary [39]. We do acknowledge the importance of longer-term follow-ups and intend to pursue the possibility of extending the follow-up period.

## 5. Conclusion

This study found significant improvements in BMI, mental health, HRQOL, weight-related body image, and physical fitness among the GASMITO patients undergoing RYGB. Psychological differences in patients with and without baseline diabetes depended on time of assessment. The diabetic group had better mental health and HRQOL on all psychological measures before surgery. However, these differences resolved around the time of surgery and no significant differences between the two groups were observed postoperatively. Thus, this study found no evidence that baseline diabetes should be considered either a positive or a negative predictor of long-term mental health and HRQOL after RYGB surgery. Furthermore, level of physical fitness (VO_2_max) as an indicator of physical activity was not a main predictor of mental health or HRQOL outcome. However, the literature is limited and further research is therefore highly needed. Future studies should prospectively investigate the long-term (>5-year follow-up) effects of type 2 diabetes and physical activity on mental health and HRQOL in a larger bariatric sample using objective methods to obtain information about weight, BMI, diabetes status, and physical activity. Specifically, physical activity is of importance as this factor is clinically meaningful and can potentially be modified. Research should therefore seek to clarify the most optimal type and intensity of physical activity that result in successful outcome with regard to both weight loss and psychological wellbeing after bariatric surgery.

## Figures and Tables

**Table 1:** Improvement of weight, BMI, physical fitness (VO_2_max), mental health (SCL-90), HRQOL (SF-36), and body image (BIQ) from baseline to time point (B), from time point (B) to time point (C), and from time point (C) to time point (D). SE: standard error; GSI: General Severity Index.

	Baseline mean (SE)	Follow-up (B) diff. (SE)	Follow-up (C) diff. (SE)	Follow-up (D) diff. (SE)
*N*	40	32	31	23
Physical measures				
Weight (kg)	127. 8 (3.0)	*−7.3 (1.2)* ^*∗∗*^	*−19.5 (1.7)* ^*∗∗*^	*−11.0 (1.8)* ^*∗∗*^
BMI (kg/m^2^)	42.7 (0.7)	*−2.1 (0.2)* ^*∗∗*^	*−7.2 (0.3)* ^*∗∗*^	*−3.4 (0.6)* ^*∗∗*^
VO_2_max (mL/min/kg)	21.1 (0.8)	0.8 (0.5)	*3.2 (0.5)* ^*∗∗*^	*3.6 (1.0)* ^*∗∗*^

SCL-90				
Somatization	1.73 (0.1)	−0.11 (0.1)	−0.005 (0.1)	*−0.17 (0.1)* ^*∗*^
Depression	1.79 (0.1)	−0.05 (1.1)	*−0.22 (0.1)* ^*∗*^	*−0.20 (0.1)* ^*∗*^
Anxiety	1.46 (0.1)	−0.02 (0.1)	*−0.15 (0.1)* ^*∗*^	−0.07 (0.1)
GSI	1.68 (0.1)	−0.06 (0.1)	*−0.13 (0.1)* ^*∗*^	*−0.16 (0.1)* ^*∗*^

SF-36				
Physical function	68.94 (3.2)	*5.95 (2.5)* ^*∗*^	*13.17 (2.9)* ^*∗∗*^	*7.68 (1.8)* ^*∗∗*^
Physical role	74.51 (4.9)	−5.27 (7.0)	*21.88 (6.3)* ^*∗∗*^	3.83 (2.7)
Bodily pain	56.25 (3.5)	4.15 (3.7)	*10.40 (3.4)* ^*∗*^	3.75 (3.3)
General health	60.89 (2.9)	5.43 (3.6)	*16.57 (2.9)* ^*∗∗*^	4.14 (3.0)
Vitality	55.10 (3.4)	*6.07 (3.0)* ^*∗*^	*11.60 (2.9)* ^*∗∗*^	*5.13 (2.3)* ^*∗*^
Social function	88.21 (2.9)	−2.53 (3.5)	1.42 (4.5)	*12.66 (4.1)* ^*∗*^
Mental role	81.00 (4.7)	−1.88 (5.9)	*16.43 (5.1)* ^*∗*^	3.95 (2.9)
Mental health	79.74 (1.9)	−0.19 (1.8)	*5.20 (1.9)* ^*∗*^	1.23 (1.9)

BIQ				
Weight-related body image	2.35 (0.1)	*0.49 (0.1)* ^*∗∗*^	*0.56 (0.1)* ^*∗∗*^	*0.27 (0.1)* ^*∗*^

^*∗*^ *p* < 0.05; ^*∗∗*^ *p* < 0.001.
